# Effectiveness of a mobile application for independent computerized cognitive training in patients with mild cognitive impairment: study protocol for the NeNaE Study, a randomized controlled trial

**DOI:** 10.1186/s13063-024-08277-5

**Published:** 2024-07-03

**Authors:** Drin Ferizaj, Oskar Stamm, Luis Perotti, Eva Maria Martin, Anja Ophey, Sophia Rekers, Daniel Scharfenberg, Tobias Oelgeschläger, Katharina Barcatta, Sigrid Seiler, Johanna Funk, Charles Benoy, Carsten Finke, Elke Kalbe, Kathrin Finke, Anika Heimann-Steinert

**Affiliations:** 1 Department of Geriatrics and Medical Gerontology, Charité—Universitätsmedizin Berlin, corporate member of Freie Universität Berlin and Humboldt-Universität Zu Berlin, Berlin, Germany; 2 Department of Neurology, Jena University Hospital, Jena, Germany; 3 Department of Medical Psychology | Neuropsychology & Gender Studies, Center for Neuropsychological Diagnostics and Intervention (CeNDI), Faculty of Medicine, University Hospital Cologne, University of Cologne, Cologne, Germany; 4 Department of Neurology, Charité—Universitätsmedizin Berlin, corporate member of Freie Universität Berlin and Humboldt-Universität Zu Berlin, Berlin, Germany; 5 Centre Hospitalier Neuro-Psychiatrique Luxembourg (CHNP), Zentrum Für Psychotherapie, Ettelbruck, Luxembourg; 6 Neuropsychological University Outpatient Clinic of the LMU Munich, Munich, Germany

**Keywords:** Mild cognitive impairment, Cognitive computerized training, Smartphone application, Randomized controlled trial, NeuroNation MED, Post-COVID

## Abstract

**Background:**

Mild cognitive impairment (MCI) involves cognitive decline beyond typical age-related changes, but without significant daily activity disruption. It can encompass various cognitive domains as the causes of MCI are diverse. MCI as well as frequent comorbid neuropsychiatric conditions like depression and anxiety affect individuals’ quality of life. Early interventions are essential, and computerized cognitive training (cCT) is an established treatment method. This paper presents the protocol for the NeuroNation MED Effectiveness Study, evaluating the self-administered mobile cCT intervention (“NeuroNation MED”) in individuals with MCI to assess training effects on cognitive domains, health competence, neuropsychiatric symptoms, psychological well-being, and the general application usability.

**Methods:**

This study protocol presents a single-blinded multicenter randomized controlled trial that will be carried out in six study centers in Germany and Luxembourg. We included adults with MCI (existing F06.7 ICD-10-GM diagnosis and TICS ≥ 21 and ≤ 32). The intervention group will use a mobile, multi-domain cCT (“NeuroNation MED”) for 12 weeks. Meanwhile, the wait list control group will receive standard medical care or no care. The eligibility of volunteers will be determined through a telephone screening. After completion of the baseline examination, patients will be randomly assigned to one of the experimental conditions in a 2:1 ratio. In total, 286 participants will be included in this study. The primary outcome is the change of cognitive performance measured by the index score of the screening module of the Neuropsychological Assessment Battery. Secondary outcomes are changes in the Cognitive Failures Questionnaire, Hospital Anxiety and Depression Scale, Health-49, Health Literacy Questionnaire, among others. All of the primary and secondary outcomes will be assessed at baseline and after the 12-week post-allocation period. Furthermore, the intervention group will undergo an assessment of the System Usability Scale, and the training data of the NeuroNation MED application will be analyzed.

**Discussion:**

This study aims to assess the effectiveness of a mobile self-administered cCT in enhancing cognitive abilities among individuals diagnosed with MCI. Should the findings confirm the effectiveness of the NeuroNation MED app, it may confer possible benefits for the care management of patients with MCI, owing to the accessibility, cost-effectiveness, and home-based setting it provides. Specifically, the cCT program could provide patients with personalized cognitive training, educational resources, and relaxation techniques, enabling participants to independently engage in cognitive training sessions at home without further supervision.

**Trial registration:**

German Clinical Trials Register DRKS00025133. Registered on November 5, 2021.

## Administrative information

**Table:** 

Title {1}	Effectiveness of a mobile application for independent cognitive training in patients with mild cognitive impairments: study protocol for NeNaE Study, a randomized controlled trial
Trial registration {2a and 2b}	German Clinical Trials Register: DRKS00025133 UTN (Universal Trial Number): U1111-1277–8721
Protocol version {3}	Version 3.0 dated 09 March 2023
Funding {4}	Synaptikon GmbH provided access to the mobile application to Charité—Universitätsmedizin Berlin free of charge and funded the conduct of the study. The study is an investigator initiated trial (IIT). The utilization of the results lies at the scientific institutions involved in the project
Author details {5a}	Charité—Universitätsmedizin Berlin, corporate member of Freie Universität Berlin and Humboldt-Universität zu Berlin, Department of Geriatrics and Medical Gerontology, Berlin, Germany Charité—Universitätsmedizin Berlin, corporate member of Freie Universität Berlin and Humboldt-Universität zu Berlin, Department of Neurology, Berlin, Germany Jena University Hospital, Department of Neurology, Jena, Germany Department of Medical Psychology | Neuropsychology & Gender Studies, Center for Neuropsychological Diagnostics and Intervention (CeNDI), Faculty of Medicine and University Hospital Cologne, University of Cologne, Cologne, Germany Centre Hospitalier Neuro-Psychiatrique Luxembourg (CHNP), Zentrum für Psychotherapie, Ettelbruck, Luxembourg Neuropsychological University Outpatient Clinic of the LMU Munich, Germany
Name and contact information for the trial sponsor {5b}	Oskar Stamm, M.Sc Charité—Universitätsmedizin Berlin | Campus Virchow-Klinikum | Augustenburger Platz 1 | 13,353 Berlin | Visiting Address: Reinickendorfer Str. 61 | 13,347 Berlin | House 7 | T + 49 30 450 553 784 F + 49 30 450 7553 784 oskar.stamm@charite.de
Role of sponsor {5c}	Study design, clinical data management, analysis, and interpretation of data; writing of the report; and the decision to submit the report for publication as per conditions laid out in the Sponsor-Investigator agreement

## Introduction

### Background and rationale {6a}

Mild cognitive impairment (MCI) is characterized by a noticeable decrease in at least one cognitive domain, surpassing what is typically expected for an individual’s age level. However, MCI does not significantly hinder a person’s ability to carry out their daily activities and the general functional independence is maintained. The impacted cognitive domains can include memory, attention, executive function, language, and visuospatial processing [1, 2]. Based on the affected cognitive abilities, MCI can be further classified as single-domain or multi-domain MCI, as well as amnestic or non-amnestic MCI [2, 3]. Single-domain MCI refers to cases where only one cognitive domain is affected, while multi-domain MCI involves the impairment of multiple cognitive domains. Amnestic MCI is the most common subtype and relates to memory impairment as the predominant feature. On the other hand, non-amnestic MCI involves impairments in cognitive domains other than memory, such as attention, executive function, or language [2]. In the International Classification of Diseases—German Modification (ICD-10-GM; [4]), MCI is classified as mild cognitive disorder under the code F06.7.

In the broader context of neurodegenerative diseases and dementia, MCI is commonly discussed and theorized as a potential precursor to dementia. However, the etiologies of MCI are multifaceted and often cannot solely be attributed to a singular cause. Instead, it encompasses a diverse array of underlying factors, including various forms of dementia such as Alzheimer’s disease, frontotemporal dementia, Lewy body dementia, and vascular dementia. Additionally, the etiological factors can be found within conditions such as Parkinson’s disease, Huntington’s disease, HIV infection, traumatic brain injury, prion disease, psychiatric disorders, substance abuse [2, 3, 5], and, more recently, the long-term and post-acute effects of COVID-19 [6–8]. The underlying etiology of MCI varies with age, where younger and middle-aged adults are more likely to have a single etiological entity, while MCI in older adults suggests a greater likelihood of degenerative or mixed etiologies [2]. This expanded perspective on MCI reveals an exceedingly heterogeneous target population that spans across all age groups. Notably, a growing number of younger individuals have been observed to experience MCI, particularly in the context of long- and post-COVID [6–8]. Henceforth, reported prevalence rates of MCI require cautious consideration, given that the prevailing body of research predominantly concentrates on older adult populations, wherein estimated prevalence rates reach up to 20% [9].

The unspecific nature of MCI’s etiologies underscores the complexity of the condition. In this context, it has been shown that MCI is frequently accompanied by neuropsychiatric symptoms such as depression or anxiety [10–13]. The presence of comorbid neuropsychiatric conditions not only exacerbates the outcomes of individuals with MCI [13, 14], but also significantly impacts their perceived quality of life [15–17]. Considering the aforementioned findings, early interventions in individuals with MCI are of utmost importance. As the core diagnostic criteria of MCI are located in impairment of certain cognitive domains, a promising intervention involves cognitive training [18–20]. Cognitive training programs aim to maintain and enhance cognitive abilities through standardized structured exercises and tasks that target one or more cognitive domains [19]. For example, the effectiveness of cognitive training was evaluated in the Advanced Cognitive Training for Independent and Vital Elderly (ACTIVE) trial. In this large-scale trial, 2832 participants, aged 65 and older, were randomly assigned to either cognitive training groups focusing on memory, reasoning, and speed of processing, or a control group. The results of the ACTIVE trial yielded improvements in the specific domains targeted by the training over a period of up to 10 years [21–23]. However, the results are not generalizable to individuals with MCI as most participants in the ACTIVE trial were cognitively healthy and the cognitive training paradigms predominantly involved supervised group sessions utilizing paper–pencil tasks. Moreover, several meta-analyses have demonstrated positive treatment effects of cCT in cognitively healthy adults [24–28].

However, over the last two decades, there were significant advancements in the development and establishment of computerized cognitive training (cCT) programs [27]. These cCT programs offer several advantages over traditional paper–pencil tasks, including enhanced interactivity, adaptability, accessibility, motivation, and adherence [29]. Moreover, these programs offer personalized training tailored to individual needs and strengths [27, 30–33]. Furthermore, cCT can be conveniently delivered via smartphone or tablet, allowing for home-administered training. This mode of delivery can have significant advantages, particularly for less mobile groups or individuals with MCI residing in rural areas.

However, the effects of cCT on individuals with MCI are heterogeneous. While there is a body of evidence that shows positive effects on global cognition [25, 31, 34–36], the effects on the underlying cognitive domains such as memory, executive function, language, and visuospatial perception are less clear [25, 36, 37]. Additionally, several meta-analyses and reviews have demonstrated consistent treatment effects on global cognitive functioning of cCT for individuals with MCI [25, 38–41]. Nonetheless, Green et al. [30] emphasized the importance of conducting randomized controlled trials to comprehensively assess the effectiveness of cCT in large samples of individuals diagnosed with MCI.

Therefore, this paper presents the protocol for the NeuroNation MED Effectiveness Study designed to evaluate the effectiveness of a self-administered mobile cCT intervention in individuals older than 18 diagnosed with MCI. Within the NeuroNation MED Effectiveness Study, the impact of an adaptive multi-domain mobile application, namely “NeuroNation MED” on a measure of global cognition will be investigated. The cCT program will be implemented independently by the participants, without further supervision through the study staff. Furthermore, this study will assess the effects of NeuroNation MED on cognitive domains, health competence, depressive and anxious symptoms, as well as psychological well-being and the general usability of the application.

## Objectives {7}

The multicenter randomized controlled study aims to investigate the effectiveness of the NeuroNation MED training app on the cognitive abilities of patients with MCI. The intervention group (IG) will utilize the app for a duration of 12 weeks, with a recommended training intensity of three sessions per week, each lasting approximately 25 to 40 min. The wait list control group (CG) can receive their regular conventional medical care prescribed by a physician or no medical care at all, but will not have access to the training app or use any other cCT during the study period.

The primary objective of this study is to determine the effectiveness of the training program in improving the cognitive abilities of individuals with MCI. Additionally, the study aims to investigate the influence of the training program on subjective cognitive functioning, health competence, depressive and anxious symptoms, and psychological well-being. Also, the usability of the provided training program will be evaluated. Furthermore, patient diaries will be utilized to gather comprehensive information regarding additional cognition-oriented lifestyle activities and therapies that are not part of the intervention.

Primary research question:

- Does a 12-week mobile self-administered cCT program improve cognitive abilities of patients with MCI?
  - H0: There is no significant difference in the index score of the screening module of the Neuropsychological Assessment Battery (S-NAB) between IG and CG.
  - H1: There is a significant difference in the index score of the S-NAB between the IG and CG, favoring the IG.

Secondary research questions:

- Does the use of the mobile cCT program lead to an improvement in depressive and anxious symptoms, subjective cognitive functioning, psychological well-being, and health-related competence in patients with MCI?
- Are there associations between the usage of the NeuroNation MED app, sociodemographic data, and changes in cognitive and psychosocial outcomes within the intervention group?
- How do participants within the IG perceive and evaluate the usability of the NeuroNation MED app?

## Trial design {8}

This study is a single-blinded multicenter superiority randomized controlled trial. The trial design for this study is a parallel group design with an allocation ratio of 2 to 1, indicating that two participants will be assigned to the intervention group for every one participant assigned to the control group. The framework for this trial is superiority, aiming to demonstrate the effectiveness of the NeuroNation MED training program in improving cognitive abilities compared to the wait list control group. The treatment context of the participants in both groups will not be influenced by the study. Participants can continue to receive standard care with all standard therapies they would normally receive outside of the study, or receive no therapy. The therapies administered will be documented using a participant diary.

All data will be collected and analyzed in a standardized manner, adhering to good clinical practice. This protocol is conducted according to the “Standard Protocol Items: Recommendations for Interventional Trials” (SPIRIT) checklist for clinical trials [42].

## Methods: participants, interventions, and outcomes

### Study setting {9}

The study will be conducted in Germany and Luxembourg, involving six research groups and universities, namely: (1) Geriatrics Research Group of the Charité—Universitätsmedizin Berlin, Berlin, Germany; (2) Neuro-Post-COVID-Center of the Department of Neurology of Jena University Hospital, Jena, Germany; (3) Department of Neurology of the Charité—Universitätsmedizin Berlin, Berlin, Germany; (4) Department of Medical Psychology | Neuropsychology & Gender Studies of the Faculty of Medicine and University Hospital Cologne, Cologne, Germany; (5) Neuropsychological University Outpatient Clinic of the LMU Munich, Munich, Germany; and (6) Centre Hospitalier Neuro-Psychiatrique, Ettelbruck, Luxembourg. Please see the clinical trials registry for the latest information on recruitment sites: https://drks.de/search/en/trial/DRKS00025133.

### Eligibility criteria {10}

The eligibility criteria were the same for all the study centers.

Inclusion criteria:

1. Age ≥ 18 years
2. Existing diagnosis of mild cognitive impairment (ICD-10-GM: F06.7)
3. Mild cognitive impairment (Telephone Interview for Cognitive Status (TICS) ≥ 21 and ≤ 32)

The determination of the specified thresholds took into account the guidelines of the TICS standardization. The following ranges and interpretation spaces were provided: unimpaired range (TICS total score: 33–41), ambiguous range (TICS total score: 26–32), range of mild impairments (TICS total score: 21–25), and range of moderate to severe impairments (TICS total score: ≤ 20). In the presence of a diagnosis of mild cognitive impairment, individuals who achieve scores in the ambiguous or mild impairment range during the screening were included in the study [43].

4. Capacity to give informed consent
5. Ability and experience in using a mobile device for app usage
6. Ability to understand German-language instructions
7. Possession of a mobile device for app usage

#### Post-COVID group

Inclusion criteria:

1. Age ≥ 18 years
2. Existing diagnosis of mild cognitive impairment (ICD-10-GM: F06.7) and post-COVID-19-Status (ICD-10-GM: U09.9) diagnosis
3. Capacity to give informed consent
4. Ability and experience in using a mobile device for app usage
5. Ability to understand German-language instructions
6. Possession of a mobile device for app usage

Exclusion criteria for all centers:

1. Severe cognitive impairment (TICS ≤ 20)
2. Presence of moderate or severe dementia
3. Legal guardianship
4. Paralysis of the dominant arm or hand
5. Visual field deficits (e.g., hemianopsia, quadrantanopia)
6. Severe, uncorrectable visual impairments (unable to visually perceive app content)
7. Severe aphasia that impairs understanding of instructions
8. Use of other apps and software (training products) that offer cognitive training

### Who will take informed consent? {26a}

The participants will receive a written informed consent form from either the study director or the designated study staff member responsible for obtaining informed consent. Before obtaining informed consent from the participants, they will be provided with detailed written information about all relevant aspects of the study. This written information will include a thorough explanation of the study procedure, outlining what kind of information will be assessed. Additionally, it will emphasize the voluntary nature of participation and the participants’ right to refuse or withdraw their consent at any time without facing any negative consequences. Any remaining questions the participants may have will be addressed to ensure their full understanding of the study before obtaining informed consent.

### Additional consent provisions for collection and use of participant data and biological specimens {26b}

As part of the informed consent process, participants will be asked to consent to the use of their data for the study’s purpose. Participants will also be requested to authorize the research team to share their data with the other study centers involved in the study. This study does not involve the collection or storage of biological samples.

## Interventions

### Explanation for the choice of comparators {6b}

The control group is a wait list control group with no dedicated intervention. After the baseline measurements, the study participants within the CG may follow their usual standard care such as occupational therapy, physiotherapy, or psychotherapy. This approach was chosen to mirror the prevailing therapeutic realities, considering that a substantial number of individuals with MCI remain untreated despite their diagnosis [44]. Subsequently, the control group will also be granted full access to NeuroNation MED after the intervention duration. Both the IG and CG will receive free access to the NeuroNation MED app for a year.

### Intervention description {11a}

The intervention includes 12 weeks of mobile adaptive and personalized cCT with NeuroNation MED. The individual exercises are performed within the NeuroNation MED app and are developed based on scientific training concepts to address the relevant cognitive domains. This entails defining the training program based on each participant’s cognitive profile and continuously adapting it according to their individual performance within the exercises. In the context of the app, the term “cognitive profile” does not refer to linking data to clinical assessments within the study, but rather to the use of in-app modules that aim to adjust the training plan and show the progress in the categories of the app. A cognitive profile is derived from relative strengths and weaknesses in the four cognitive domains defined by the NeuroNation MED app (i.e., psycho-motor speed, reasoning, attention, memory). Therefore, these in-app modules are aligned with the exercise design of the app. For the various performance assessments in the app, which form the basis of the personalization algorithm, reference values from the NeuroNation App database, which are available aggregated per age group/exercise within the app/in-app modules adjusting the training plan, are used. Furthermore, the NeuroNation MED app offers supplementary content (“NeuroBooster”) providing participants with the option to access 19 different resources on health literacy, performance enhancement, well-being, and relaxation. These resources feature graphical instructions with short animations to demonstrate the correct execution of the exercises.

Participants within the IG can choose from four intensity levels ranging from 25 to 40 min. Trial participants are recommended to undergo a 12-week training program, consisting of three training sessions per week. Each exercise has a net time of 90 s with the net time being the duration the exercise timer is active. However, training sessions can exceed 40 min due to the need for extra time required to work through explanatory texts, educational content, and user guidance.

### Criteria for discontinuing or modifying allocated interventions {11b}

Each participant of the study will be informed that participation is voluntary and that withdrawal from the study is possible at any time. It will be communicated that withdrawal will have no effect on possible further treatment at any participating institution. The study may be terminated before its intended completion if ethical concerns arise, if there is insufficient recruitment of participants, or if the safety of the participants is compromised or uncertain. Furthermore, termination may occur if changes in accepted clinical practices render it unwise to continue the clinical trial, or if early indications of harm from the intervention are observed.

Since the study follows an intention-to-treat approach, participants in the IG will not be prescribed a fixed amount of training time or session count for using the NeuroNation MED app. At the first appointment, the IG is instructed to adhere to a certain weekly usage duration and frequency of use, but this is not mandatory for study participation and may vary among participants during the intervention period.

### Strategies to improve adherence to interventions {11c}

In order to increase the adherence of the participants in the intervention group, various actions are implemented. Firstly, all participants in the IG receive a printed manual with instructions on how to use the NeuroNation MED app. This includes information on how to use the platform, troubleshooting solutions, contact persons, and recommended training duration and intensity. Additionally, the participants receive a weekly newsletter via an anonymous mailing list with tips on a healthy, active lifestyle, psychoeducational content regarding cognitive and brain plasticity, and motivational content. Exemplary topics of the 12 distinct newsletters are risk and protective factors of cognitive health, emotional well-being, the Mediterranean diet, physical activity, and social networks. After the first appointment, the participants of the IG receive an email containing their access credentials and helpful information about the NeuroNation MED app. Another follow-up email will be sent 1 week after t_0_, serving as a reminder and inquiring about any technical problems or if the training has commenced.

### Relevant concomitant care permitted or prohibited during the trial {11d}

Study participants in both study groups will be asked not to perform any other cCT program (other than the intervention) as part of their study participation. However, all participants were allowed to follow any other forms of therapy in the context of medical treatment (e.g., ergotherapy, medication, etc.) or private measures (sports, social activities, etc.). Rather, these should be documented by the participants of both the CG and IG in a patient diary, which should be continuously updated during the intervention period of 12 weeks.

### Provisions for post-trial care {30}

No direct relevant risks arise for the study participants from the implementation of the training units within the app or the study-related assessments or questionnaires. All participants will be granted complimentary 12-month access to the NeuroNation MED app upon completion of the study.

### Outcomes {12}

The primary endpoint of the study is the S-NAB adapted to German language [45, 46]. As a comprehensive test battery for neuropsychological assessment, the S-NAB is primarily used in clinical neuropsychology with adults aged 18 and older. The S-NAB combines 14 individual neuropsychological tasks and thus offers the possibility of gaining a diagnostic overview in a short time frame. The functional areas of attention, language, memory, perception, and executive functions are examined with at least two tasks each. Due to its short duration, the S-NAB module is well-suited for patients with limited resilience. Compared to other neuropsychological screenings, the tasks of the S-NAB are challenging enough to detect mild to moderate cognitive impairment [47]. Furthermore, the S-NAB is available in two parallel versions, which is a necessity for the pre-post design of the current study. The S-NAB offers norm scores covering the entire adult age range based on a representative sample from Germany. Additionally, the S-NAB has a mean score of 100 with a standard deviation of 15.

Several important psychosocial and health-related aspects were selected as secondary endpoints. Therefore, in the current study, the following constructs were recorded: psychological well-being, self-efficacy, depressive symptoms, and anxiety symptoms.

The Hamburg Modules for the Assessment of General Aspects of Psychosocial Health for Therapeutic Practice (HEALTH-49) is a self-report instrument for the multidimensional assessment of psychosocial health [48]. This instrument takes into account psychosocial aspects in therapy planning and diagnostics, as well as in quality assurance and the evaluation of psychotherapeutic and medical treatments in general. For the current study, the two modules Psychological Well-Being (5 questions) and Self-Efficacy (5 questions) of the HEALTH-49 were selected. Each module is rated on a Likert-type scale ranging from zero to four, with total scores that are averaged. Additionally, lower scores on both subscales are indicative of elevated levels of self-efficacy and general well-being.

The Hospital Anxiety and Depression Scale—German Version (HADS-D) is a medical psychological questionnaire and is used to assess anxiety and depressive symptoms in patients with physical illnesses or physical complaints [49–51]. The extent of anxious and depressive symptoms during the past week is assessed by means of self-report, which is recorded on two Likert-type subscales with seven items each that range from zero to three. Higher total scores on the subscales indicate higher levels of anxiety or depressive symptoms. The cumulative score of the two subscales provides a measure of general psychological impairment.

Especially in the context of neurological diseases, a significant measure of quality of life involves the subjective impairment resulting from the disease’s impact on cognitive failures in daily life. The Cognitive Failures Questionnaire—German Version (CFQ-D [52]) is a self-assessment questionnaire consisting of 32 Likert-type items. It evaluates the frequency of committed everyday errors in the past year across the domains of perception, memory, and action regulation [53, 54]. The items are scored on a scale from zero to four. By summing up all the item scores, the total score is derived, which serves as a measure of an individual’s inclination to make everyday errors. Higher total scores indicate a higher subjective probability of encountering everyday mistakes and cognitive lapses.

Health literacy and patient sovereignty are potential influencing factors on patient health, as higher competence and sovereignty in this area is associated with both higher treatment adherence and better preventive health behaviors [55]. Measurement of this aspect is crucial, serving as both a control variable to ensure comparability between the intervention and control groups, and as a means to detect potential positive effects of the intervention on this aspect. The Health Literacy Questionnaire—German Version (HLQ-G) [56, 57] was selected as the measurement instrument. The questionnaire comprises 44 questions distributed across nine domains, with each domain containing 4 to 6 questions. The domains capture (1) feeling understood and supported by health care providers, (2) having enough information to manage one’s own health, (3) actively doing something for one’s own health, (4) social support for one’s health, (5) evaluating health information, (6) ability to actively engage with health care providers, (7) navigating the healthcare system, (8) ability to find good health information, and (9) understand health information well enough to apply it. For the first five subscales, the scores range from 1 to 4, while for scale six to nine, the scores range from 1 to 5. Higher scores reflect higher health literacy. To obtain scores for each domain, the response to the questions are summed and averaged.

In order to analyze the usability of the NeuroNation MED app, the System Usability Scale (SUS) is used [58]. This is a widely used questionnaire developed to assess the usability of a system, product, or service. It consists of 10 items on a 5-point Likert scale that measures the user’s perception of various aspects of usability, such as ease of use, learnability, efficiency, and satisfaction. The SUS provides a standardized and reliable method to evaluate and compare the usability of different systems. The SUS score ranges from 0 to 100 and can be interpreted in many ways, e.g., by percentiles, grades, acceptability, adjectives, or the Net Promoter Score. The raw SUS values can be converted into percentile ranks. The average value at the 50th percentile is 68.

Moreover, usage data from study participants will be recorded for analysis. This includes documenting both the frequency of training days and the duration of app usage. To effectively account for potential confounding variables during the survey period and to evaluate the impact of external factors on participants’ cognitive status, participants will maintain a study diary throughout the 12-week duration. This diary aims to document activities that have the potential to influence cognitive status.

Exclusively at the test center of Jena University Hospital (JUH), further secondary outcome measures encompass visual attentional parameters based on the theory of visual attention [59], assessed in a whole report paradigm. In addition, clinical routine data relevant to post-COVID syndrome will be measured, such as questionnaires concerning fatigue (Brief Fatigue Inventory [60], Fatigue Assessment Scale [61]), psychological burden (Patient Health Questionnaire 9 [62], Beck Depression Inventory II [63], Post-Traumatic Stress Syndrome 14-Questions Inventory [64]), sleep/sleepiness (Pittsburgh Sleep Quality Index [65]; Epworth Sleepiness Scale [66]), cognition (Functional Assessment of Cancer Therapy—Cognitive Function [67]), and established neuropsychological tests (computerized Test of Attentional Performance—TAP [68]).

### Participant timeline {13}

The complete participant timeline is illustrated in Table 1.

**Table 1:**
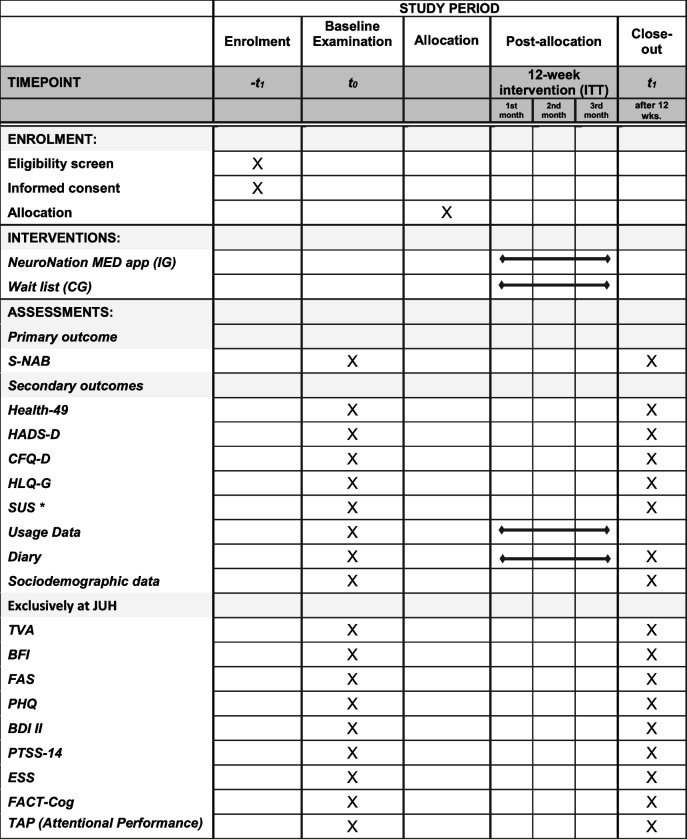
SPIRIT schematic schedule of enrolment, interventions, and assessments for the NeuroNation MED trial

*Only applied in the IG.S-NAB: Screening Module of the Neuropsychological Assessment Battery; HADS-D: Hospital Anxiety and Depression Scale (German Version); HEALTH-49: Hamburg Modules for the Assessment of Psychosocial Health in Clinical Practice; CFQ-D, Cognitive Failures Questionnaire; HLQ-G: Health Literacy Questionnaire (German Version); SUS: System Usability Scale; Usage Data, completed exercises, duration of use, number of training days etc.; Diary: patient diary with activities that could have an effect on cognitive status. Exclusively at the test center of JUH, further secondary outcome measures include visual attentional parameters based on TVA; BFI: Brief Fatigue Inventory; FAS: Fatigue Assessment Scale; PHQ: Patient Health Questionnaire 9; BDI II: Beck Depression Inventory II; PTSS-14: Post-Traumatic Stress Syndrome 14-Questions Inventory; ESS: Epworth Sleepiness Scale; FACT-Cog: Functional Assessment of Cancer Therapy—Cognitive Function; TAP (Attentional Performance): computerized Test of Attentional Performance

### Sample size {14}

The assumed effect size is based on results of previous studies using the cognitive training program “NeuroNation BT” [69] and comparable digital cognitive training applications [25, 26, 36, 70]. A calculation by means of a *t*-test for independent samples was used as the basis for the sample size calculation within the trial described here. With an allocation ratio of 2:1, if 164 subjects are included in the IG and 82 subjects are included in the control group, differences with an effect size of 0.39 or greater can be shown (a priori power analysis, Wilcoxon-Mann–Whitney test (two groups), power = 80%, two tails significance level *α* = 0.05). This resulted in the total sample size of 246. We expect a drop-out rate of approximately 15% [71], which results in a sample size to be recruited of *N* = 286. This sample size calculation was performed with G*Power 3.1.

### Recruitment {15}

Potential participants are recruited via the volunteer database of the Geriatrics Research Group of the Charité—Universitätsmedizin Berlin, the Neuro-Post-COVID Center of the Department of Neurology of Jena University Hospital, the Department of Neurology of the University Hospital Cologne, the Clinic for Neurology with Experimental Neurology, Charité, and the Neuropsychological University Outpatient Clinic of the LMU Munich. At the Centre Hospitalier Neuro-Psychiatrique in Luxembourg, participants were recruited from the long-COVID consultation. Furthermore, (neuro-) rehabilitation clinics, psychotherapists in private practice, and various clinics of the Charité and the other participating study centers will be used as gatekeepers for recruitment and flyers will be distributed. In addition, announcements on intranet sites will be used, a newsletter will be sent to NeuroNation users, and outdoor and radio advertising will be used to draw attention to the study.

The planned study will include diagnosed patients with cognitive impairment (ICD-10-GM: F06.7). The study population will be recruited via the clinics of the study centers involved in the study and via external practices as multipliers, each of which is diagnosed as part of standard care. These clinics and practices will approach patients with diagnoses and characteristics matching the inclusion criteria for the planned survey. The multipliers receive flyers designed for the study that provide information on the inclusion and exclusion criteria applicable to this study. These also include, by way of example, the diagnosis relevant to this study. Thus, all potential study participants have a medically confirmed ICD-10-GM F06.7 diagnosis.

## Assignment of interventions: allocation

### Sequence generation {16a}

The randomization process in this study incorporated permuted block sizes (using block sizes that enable a 2:1 allocation), which were generated by the research coordinator of every study center. This approach aimed to maintain allocation concealment and achieve balanced participant allocation across the treatment groups, minimizing potential bias in the study.

### Concealment mechanism {16b}

To avoid selection bias, t_1_ (12 weeks after the baseline measurement) will be conducted by a different study staff member not involved in t_0_ (randomization and introduction to the training). Once baseline measurements are completed for a participant, a staff member will randomize the participant by a manual procedure using urn sampling without replacement and will not be allowed to conduct t_1_. The allocation is entered by the research coordinator of the study center into a password-protected Excel file, to which the study staff has no access. This procedure ensures that at t_1_, the research team is unaware of which treatment group a participant has been assigned to.

### Implementation {16c}

Randomization will be performed on the patient level with a 2:1 ratio using block randomization with varying block sizes, stratified per sex. Participants can be enrolled only by trained study personnel. For the intervention group, the manual and login voucher will be handed out after randomization. Subsequently, the study staff installs the app with the participants, registers them, and gives instructions on the frequency and duration of the training.

## Assignment of interventions: blinding

### Who will be blinded {17a}

Study participants in the IG will participate in an app-supported cCT program over a 12-week period, while the control group will receive standard medical care or no care. Consequently, participants will be aware of their group assignment. Thus, this design prevents blinding of the study participants of their group allocation. Thus, the study will be conducted in a single-blinded design. The two study appointments will be carried out by members of the study staff, who will be blinded to the group assignment of the respective participants at both visits. Participants will be instructed before the start of the second appointment not to make any reference to their group allocation. The blinding will not be undone until the end of the study appointment. After the second appointment, participants in the control group will be given access to the NeuroNation MED app.

### Procedure for unblinding if needed {17b}

Unblinding of the study personnel will only happen in case of serious adverse events; circumstances for medical intervention not permitted by the study protocol; intercurrent illness/infection; other significant protocol violations; personal request of the study participant (withdrawal of patient consent); and any other situation where, in the opinion of the principal investigator, continued study participation would not be in the best interest of the subject. If serious adverse events occur during the trial or performance evaluation, they must be reported to the sponsor.

## Data collection and management

### Plans for assessment and collection of outcomes {18a}

The t_0_ and t_1_ take place in the facilities of the study centers and the associated examination rooms. A Trial Master File (TMF) documents all essential communication and other processes.

The collected data from the questionnaires/assessments will be recorded in paper form and filed accordingly in the Investigator Site File (ISF). Additional notes are logged on paper as part of the data backup process and later digitally recorded. Logging data of the NeuroNation MED app usage will be provided to the study centers. Personal data documented on paper (e.g., consent forms, patient and participant files) must be stored in lockable cabinets. The allocation of keys must be organized and controlled within the study team. The allocation of keys must be documented. Controlled key distribution should ensure that only authorized study team members have access to the data. Keys are not to be given to non-authorized individuals. Lockers are to be kept locked at all times. When an employee leaves, the corresponding key must be returned immediately.

Access control to the premises is only possible for authorized persons, key allocation or transponder system.

The corresponding data storage takes place in a secured folder within the servers of the study centers. The folders are only accessible to the members of the research team. Further access requires the active activation by the rights holder of the folder. The access rights within the study team are regulated by the head of the study team; any change of these rights requires his approval. Electronically maintained participant identification lists must be secured by a password. All study data (paper and digital) will be deleted after the legally prescribed retention period of 10 years has expired.

### Plans to promote participant retention and complete follow-up {18b}

For participants who withdraw consent for any reason, their previously collected data will not be used for analyses. If participants drop out of the study or have missing data, data imputation will be performed. Moreover, if participants deviate from the intended intervention (usage of the application), the initially scheduled follow-up visit will still be conducted and the data will be utilized.

### Data management {19}

The procedure for data entry, collection, and storage is described in detail below.

Technical organizational measures:

- Pseudonymization of data in password-protected Case Report Form (CRF; in an Excel file).
- Access control to the premises (concerns offices for storage of paper documents) is only possible for authorized persons and is regulated by key allocation.
- In addition to the use of the app, the therapy also includes psychoeducational materials, which are sent to the participants by mail from the manufacturer’s side. In order to protect the personal data of the users and in particular to prevent the contact email addresses from being passed on outside the study centers, the centers create mail distribution lists in which the addresses of the test persons are stored. The app manufacturer has no access to this and only sends the materials to the general distribution list and has no insight into the mail addresses behind it.

Access to study-specific electronic files: PC workstations of the respective centers, server infrastructure, authorization system, password-protected access to the personal account.

- Data transfer within the study centers in pseudonymized form and externally in anonymized form.
- Regular automatic backup of study data is performed routinely.
- Description of the documentation procedure.

The collected data from the introductory questionnaires are recorded in paper form and filed accordingly in the ISF. Additional notes are logged on paper as part of the data backup process and later digitally transcribed.

The corresponding data storage is done in a secured folder within the network of the respective study centers. These folders are only accessible to the study staff of the respective center. Further access options require active activation by the rights holder of the folders. The access rights within the study team are regulated by the study manager; any change of these rights requires his approval.

In addition, study data is deleted after the legally prescribed retention period of 10 years has expired. Subject-related data will be collected in pseudonymized form. All subjects are unmistakably identified by a participant number, assigned upon registration. The study directors of each study center maintain a confidential participant list, in which the identification data are linked to the full subject name, to which only he and one other member of the study staff have access. Questionnaires will be stored in a lockable cabinet at the study center.

Paper records:

- Storage location patient file, study file, CRF: premises of the respective study centers.
- Restricted access room, lockable cabinets.
- It is not possible to trace CRF data to individual patients without an identification list.
- Only the study directors of each study center have access to the identification list of their participants.

### Confidentiality {27}

The questionnaires and assessments will be recorded in paper format and stored in the Investigator Site File (ISF). Additional notes will be documented on paper and transcribed digitally at a later stage. The data will be saved on secure folders within the network of each study center. These digital folders will only be accessible to the study personnel of the respective center. Any additional access requires active authorization from the folder’s rights holder. Further, the access permissions within the study team are regulated by the principal investigator, and any changes to these permissions require their approval. Furthermore, the study data will be deleted after the legally mandated retention period of 10 years. Participant-related data will be collected in a pseudonymized form where every participant will be uniquely identified by a two-digit number during registration. The study directors maintain a confidential participant list, which associates the identification data with the full participant name. This participant list is only available to the study directors and one other member of the study personnel. The paper-based questionnaires and other collected data will be kept in a lockable cabinet at the respective study center.

### Plans for collection, laboratory evaluation, and storage of biological specimens for genetic or molecular analysis in this trial/future use {33}

This is not applicable as no biological specimens or genetic material has been collected in the present study.

## Statistical methods

### Statistical methods for primary and secondary outcomes {20a}

The statistical analysis will be conducted after the last t_2_ of the last patient. Collected data from assessments, questionnaires, and usage logs will be processed and cleaned in Excel. Subsequently, the data will be analyzed using statistical programs such as SPSS, R-Studio, STATA, and Python 3.9 with statistical libraries including dplyr, ggplot2, car, NumPy, Pandas, Pingouin, and Matplotlib. Both metric and non-metric variables will be considered in the statistical analysis.

The data from the IG and CG will be initially analyzed descriptively using sociodemographic data to describe the sample, assessing the baseline status of the participants and evaluating the assessment after 12 weeks. The following descriptive statistics will be reported depending on the measurement level of each variable: frequencies (absolute and/or percentage), available and missing data counts, mean, standard deviation, standard error, lower and upper quartiles, minimum and maximum values as well as confidence intervals. Graphical representations of key variables will be generated using scatter plots, histograms, box plots, mean value plots, or other common graphics, based on the measurement level of the variables. Missing data will be included in these analyses. Therefore, datasets classified as missing not at random (MNAR) will be imputed using sensitivity analyses and a pattern mixture model, and considered further in subsequent statistical analyses.

To achieve comparability between the experimental groups at baseline, the IG and CG will be compared in terms of sociodemographic characteristics. The independent samples Welch’s *t*-test will be used for normally distributed metric variables, while the chi-square test will be employed for categorical variables. If a normal distribution is not found in the data collected, the Wilcoxon signed-rank test or Mann–Whitney *U* test will be applied. ANCOVA will be conducted to evaluate the training effects between the IG and CG, using the baseline values as a covariate and incorporating gender, experimental group (IG and CG) as independent variables, and the post-assessment score as dependent variable. Additionally, age and study center will also be examined as covariates. If the data fail to meet the assumptions for performing parametric ANCOVA, non-parametric tests such as the Wilcoxon test or Mann–Whitney *U* test will be applied instead. Effect sizes and confidence intervals will be calculated for the respective statistical tests to enhance result interpretability.

Using the logging data from the NeuroNation MED app, Pearson correlations will be used to examine the relationships between the number of training days, completed training units, and changes in assessments within the IG. Furthermore, one-way ANOVA will be employed to investigate potential associations between usage frequencies and sociodemographic data.

### Interim analyses {21b}

An interim analysis will be conducted when the sample size reaches 50 participants. Employing the alpha-spending function, which is a commonly used method for interim analysis in clinical trials, the alpha level was determined to be 0.00305 for the interim analysis and 0.04695 for the final analysis of the primary outcome. The alpha level for the secondary outcomes in the final analysis will be adjusted to 0.00783 using the Bonferroni correction. Also, the sociodemographic data of the participants that were included in the interim analysis will be compared with the total sample using a multivariate ANOVA with the factor participant group (interim analysis vs. remaining participants). The study will be terminated in the event of severe adverse effects that demonstrate noticeable or statistically significant declines in the primary and secondary outcomes.

### Methods for additional analyses (e.g., subgroup analyses) {20b}

The primary endpoint analysis will be supplemented by three exploratory subgroup analyses based on age (< 65 years, ≥ 65 years), MCI subtype based on the baseline cognitive assessment values (amnestic MCI, non-amnestic and multi-domain MCI), and gender (male, female). The results of all subgroup analyses will be presented in a combined table and a forest plot, including effect size estimators (η^2^) and a 95% confidence interval (CI). The table will also include the number of patients in each respective subgroup and the *p* values. The explorative statistical analysis of secondary outcomes in the post-COVID group (see {12}) follows, where appropriate, the outlined approach for the primary endpoint, see {20a}.

Furthermore, the S-NAB scores and CFQ-D values at baseline and follow-up will be standardized using *z*-scores to determine cognitive performance estimation accuracy. The average discrepancy scores between groups will be analyzed using *t*-tests and correlational analysis.

### Methods in analysis to handle protocol non-adherence and any statistical methods to handle missing data {20c}

Excluding participants with missing values from the analysis can bias the statistical analysis. Hence, a multiple imputation approach is conducted. Missing value information will be gathered through follow-up telephone interviews when a participant withdraws from the study. These interviews will assess the reasons for discontinuation.

Within this study, all of the missing data will be imputed. This process will involve the generation and pooling of multiple complete datasets, where missing values are estimated based on the observed data. To assess the robustness of our imputations and the potential impact of missing data on the study’s primary outcome, sensitivity analysis will be conducted using delta adjustment. Moreover, an intention-to-treat analysis will ensure that every participant who took part in the baseline assessment is included in the final analysis. This includes the IG, regardless of their adherence to the proposed training protocol.

### Plans to give access to the full protocol, participant-level data, and statistical code {31c}

Regularly, safety reports are generated and distributed to the Data Safety and Monitoring Committee. The datasets analyzed during the current study will be available from the management committee after the primary publication on reasonable scientific request.

## Oversight and monitoring

### Composition of the coordinating center and trial steering committee {5d}

All lead investigators of the study centers are the trial steering committee members. Within the study, regular internal monitoring is performed by the study directors of the various study centers. It will be observed and documented that the ethical and regulatory framework conditions previously defined by the Charité are taken into account. Furthermore, it will be ensured that the data collected in the course of the study will be properly and accurately recorded, stored, processed, and reported. The data processed must be checked regularly for plausibility and completeness and corrected and/or supplemented if necessary. This is done within the study centers according to the dual control principle in data entry and with range checks of values before data analysis. Throughout the study, applicable guidelines of good clinical practice are considered and compliance with these is monitored. The defined times of monitoring are at the beginning of the study (first patient in), after 6 weeks, and at the end of the study period (last patient out). The trial steering committee (TSC) is composed of lead investigators of the study centers. They or their representatives will meet over the course of the trial at least bimonthly to oversee the conduct and progress of the trial.

### Composition of the data monitoring committee, its role and reporting structure {21a}

Data monitoring committee (DMC) is not needed. The trial involves a low-risk intervention and the potential adverse effects are well-understood. Previous studies have shown no significant adverse events or serious safety concerns related to this intervention. Therefore, the likelihood of unexpected safety issues arising during this trial is minimal.

### Adverse event reporting and harms {22}

Adverse events (AEs) are defined as any undesirable or unintended signs, symptoms, or medical occurrences that happen to participants during the course of the study, which does not necessarily have a causal relationship with the investigational product. All serious adverse events must be reported to the principal investigator within 24 h.

A serious adverse event is defined as any event that:

- Death
- Life-threatening illness or injury
- Permanent impairment of a body structure or function
- Hospitalization or prolongation of existing hospitalization
- Medical or surgical intervention to prevent permanent impairment of a body structure or function
- Damage to a fetus, fetal death, congenital malformation, or birth defect

All adverse events occurring during the study will be recorded on an AE/SAE Report Form. The AE/ SAE report includes details of the report, details of the person concerned, description of the incident, and relation between incident and participation in the study.

### Frequency and plans for auditing trial conduct {23}

A quality assurance audit/inspection of this study may be conducted by BfArM as part of the fast-track process for the digital health application (DiGA). Each study center has the right to verify compliance with the cooperation agreement on the part of the respective other study center if this is necessary to fulfill an obligation to a supervisory authority or to satisfy itself that the respective other study center has adapted its operations to the provisions of this agreement following a data protection incident. If and to the extent that such review requires the performance of on-site inspections, such inspections shall usually take place during normal business hours and without unnecessary disruption of operations. The party conducting an inspection shall give the other party reasonable advance notice of all circumstances related to the inspection. A study center may commission a third party to conduct the review. In such a case, the third party shall be obligated in writing to maintain secrecy and confidentiality, unless the third party is subject to a professional duty of confidentiality.

### Plans for communicating important protocol amendments to relevant parties (e.g., trial participants, ethical committees) {25}

The study has been prospectively registered with the German Clinical Trials Register under the registration number DRKS00025133 (11/05/2021). In the event of any significant modifications to the study protocol, a formal submission will be made to the Ethics Committee for approval prior to implementing the changes. Also, participants who have already signed informed consent will be notified about the modifications through email or letter. Moreover, all amendments will be recorded in the German Clinical Trials Register and the respective study documents including informed consent and written study information will be updated accordingly.

## Dissemination plans {31a}

After completion of the analysis of the data set, the results will be published in scientific journals in several publications with focus on different research questions by the members of the research centers. Furthermore, results will be presented at national and international conferences. All persons involved in the recruitment, study execution, analysis, and organization of the research project will be considered as co-authors.

## Discussion

In the past two decades, there has been a surge in mobile cCT programs aimed at improving cognitive functioning. Substantial evidence has demonstrated that cCT leads to improvements in specific cognitive domains that are subject to training [27]. However, the conceptualization and operationalization of cCT remains heterogeneous, with approaches ranging from standard psychological tasks to gamified variants and commercial video games [30]. Recent research suggests gamified cCT programs confer a higher degree of engagement, motivation, and cognitive demand to non-gamified counterparts [29]. As such, adherence rates in the present study may remain high despite utilization of a real-word approach without additional supervision through the study staff. The focal research question of this study pertains to the effectiveness of a clearly delineated mobile cCT for a specific target population, namely individuals with MCI. This approach, utilizing gamified multi-domain cCT and a definitive objective, aligns with best practices in cCT intervention research [30]. In summary, this highlights the need for the NeuroNation MED Effectiveness Study, a multicenter randomized controlled trial to evaluate the effectiveness of the NeuroNation MED application. Furthermore, in Germany, accessing cognitive training through memory consultations often involves long waiting times and requires sufficient mobility and time from those affected. These barriers hinder adequate health care for people with MCI, which can be efficiently addressed with the help of mobile cCT. Green et al. [30] provided methodological and theoretical guidelines for assessing the effectiveness of cCT programs in real-world scenarios. Our study aims to address this need by examining the impact of a mobile home-administered cCT program in individuals with MCI. A notable aspect of our study is the focus on mobile cCT, which offers advantages such as cost-effectiveness, enhanced accessibility, flexibility, and potential for greater adherence compared to traditional supervised or computer-based training methods. This emphasis serves as a bridge between controlled laboratory-derived findings and their practical utility in real-world scenarios. To achieve this, we utilized an intention-to-treat analysis of the collected data. This approach ensures that all participants are included in the analysis, regardless of adherence, providing a more robust assessment of the intervention’s effectiveness in real-world conditions. Additionally, our study addresses the lack of high-quality RCTs with sufficient statistical power [30]. By focusing on mobile training and using an intention-to-treat analysis, we aim to provide insights regarding effectiveness on cognitive endpoints as well as neuropsychiatric symptoms, usability, and participant engagement.

## Trial status

The trial status is ongoing with the first patient recruited in August 2021. We expect the recruitment to be finished by August 2023 and the last patient out will be in November 2023. The current protocol is version 3 dated 09 March 2021.

## Data Availability

The datasets of the study will not be publicly available so that the anonymity of participants involved may be preserved. The dataset may be available from the corresponding author on reasonable scientific request.

## Abbreviations

ACTIVE: Advanced Cognitive Training for Independent and Vital Elderly
AE: Adverse events
ANCOVA: Analysis of covariance
cCT: Computerized cognitive training
CeNDI: Department of Medical Psychology | Neuropsychology & Gender Studies, Center for Neuropsychological Diagnostics and Intervention
CG: Control group
CFQ-D: German version of the Cognitive Fusion Questionnaire
CHNP: Centre Hospitalier Neuro-Psychiatrique Luxembourg
CI: Confidence interval
CRF: Case Report Form
DMC: Data monitoring committee
DiGA: Digital health application
HADS-D: Hospital Anxiety and Depression Scale—German Version
HEALTH-49: Hamburg Modules for the Assessment of General Aspects of Psychosocial Health for Therapeutic Practice
HLQ-G: Health Literacy Questionnaire—German Version
ICD-10-GM: International Classification of Diseases—German Modification
IG: Intervention group
IIT: Investigator initiated trial
ISF: Investigator Site File
JUH: Jena University Hospital
MAR: Missing at random
MCAR: Missing completely at random
MNAR: Missing not at random
MCI: Mild cognitive impairment
RCT: Randomized controlled trial
S-NAB: Screening Module of the Neuropsychological Assessment Battery
SPIRIT: Standard Protocol Items: Recommendations for Interventional Trials
SUS: System Usability Scale
TICS: Telephone Interview for Cognitive Status
TMF: Trial Master File
TSC: Trial steering committee
UTN: Universal Trial Number
